# LKB1 in transmembrane receptor signaling

**DOI:** 10.18632/oncotarget.4777

**Published:** 2015-07-01

**Authors:** Imoh S. Okon, Ming-Hui Zou

**Affiliations:** Center for Molecular and Translational Medicine, Georgia State University, Atlanta, GA, USA

**Keywords:** LKB1, transmembrane receptors, signal transduction

Signal processing and integration is critical in physiology, as it is in disease conditions. Conversion of extracellular signals into appropriate biological responses by cell membrane receptors, and subsequent extinction of signaling events is critical to cellular hemostasis. Defective trafficking, internalization or degrardation of transmembrane receptors (TMRs) result in perturbed activation of signaling networks, which play essential roles in disease initiation or accentuation. In our recent study, we found that liver kinase B1 (LKB1), a calcium–calmodulin family member abrogated neuropilin-1 (NRP-1) protein in lung cancer clinical specimens and cell lines [1]. Surprisingly, this observation was reminiscent to our previous finding in which LKB1 attenuated the activation of a repertoire of receptor tyrosine kinases (RTKs), including EGFR, ErbB2, HGFR (c-Met) and EphA2 [2]. The regulation of TMRs by LKB1 appears to be a recurring pattern in cancer cells but occur via different mechanisms. Unlike LKB1-mediated dephosphorylation of RTKs via accentuation of selected phosphatase activity, NRP-1 attenuation was promoted by LKB1-RAB7 GTPase complex [1, 2]. We demonstrated for the first time that LKB1 is a RAB7 effector and suppresses tumor angiogenesis by promoting cellular trafficking of NRP-1 from RAB7 vesicles to the lysosome for degradation. LKB1 specifically bound active GTP-Q67L RAB7 construct, but not the dominant-negative GDP-T22N form. NRP-1 localization within RAB7, a late endocytic target was not detected in early (RAB5) or recycling (RAB11) endosomal markers [1]. With over 60 different mammalian RAB family-members, the selectivity may be context-dependent, and the relationship between LKB1 and RAB family-members requires further investigation. Our findings indicated that hypoxia, a property of tumor micro-environment promotes LKB1 nuclear export to the cytosol, where it interacts with NRP-1 and RAB7 [1]. LKB1 expression was consistent with its tumor suppression functions, which included inhibition of angiogenesis and tumor growth *in vivo*. Conversely, loss of LKB1 exacerbated tumor-enhancing phenotypes [1]. We have recently observed a similar inverse expression profile between LKB1 and NRP-1 proteins within endometrial cancer specimens. We found that NRP-1 positively correlated with NEDD9 pro-metastatic protein. As expected, LKB1 abrogation of NRP-1 protein correlated with decreased metastatic potential of cancer cells *in vitro* (unpublished observations).

In contrast to well-established RTKs, such as EGFR that have been studied for decades, several NRP-1 functions remain relatively unknown. NRP-1 is a non-tyrosine kinase, type-I transmembrane receptor that is largely associated with the VEGF receptor family. Canonical NRP-1 functions in tumor angiogenesis is strongly linked to VEGFR2. In our studies, NRP-1- mediated tumor phenotypes were independent of VEGFR2 in lung and endometrial cancer specimens [1]. Angiogenic-switch which is characterized by heightened development of new blood vessels, serve to enhance nutrition and oxygen availability required for tumor progression, and NRP-1 may be critical to the process. LKB1, as a key regulator of TMRs influences receptor activation, trafficking, internalization, recycling and degradation. As expected, the attenuation of TMR-mediated signaling events impact biological and functional outcomes, such as tumor angiogenesis and migration. We have found decreased recycling potential in LKB1-positive cells relative to controls (LacZ) following ligand stimulation at different time intervals. We also observed that LKB1 associates with another GTPase protein, dynamin2 (unpublished observations), suggesting that LKB1 may possess TMR trafficking functions via selective complex formation with GTPase family members.

LKB1-mediated targeting of TMRs appear to be specific and highly selective. For example, NRP-2, an isoform of neuropilin receptors was unaffected by LKB1. Unlike NRP-1, NRP-2 protein was stable under varying conditions, including hypoxia. This observation is consistent with a recent report that described NRP-1 attenuation under starvation compared with robust NRP-2 expression [3]. The reasons for these differences are unclear, however both receptors share only a 44% sequence homology which may account for differential regulation with respect to LKB1. These differences may also explain divergent functions in development and disease. Although both are important in the vascular system, NRP-1 is widely linked with tumor angiogenesis while NRP-2 is associated with lymphangiogenesis [4]. Interestingly, LKB1 failed to abrogate TMRs at the message level, suggesting posttranslational modifications which may include, acetylation, phosphorylation or farnesylation. LKB1 possesses a farnesylation site at the C-terminal domain, and potential functions of the domain remains to be explored. At the last count, LKB1 modulatory functions involved approximately 13 downstream substrates, including the well-described energy sensor, AMP-activated protein kinase (AMPK). Such orchestrated regulation by LKB1 may directly modulate several signaling networks or occur indirectly via these substrates. Although several roles have been ascribed to LKB1 in the last decade, ranging from hematopoietic stem cell survival, tumor suppressor and cardiovascular functions [1, 5–7], it appears that the ubiquitously expressed *LKB1* gene has yet to play more critical roles in disease and physiology.
